# Phenotypic drift in metastatic progression of breast cancer: A case report with histologically heterogeneous lesions that are clonally related

**DOI:** 10.1002/ccr3.3257

**Published:** 2020-08-27

**Authors:** Jamie R. Kutasovic, Amy E. McCart Reed, Anna Sokolova, Janani Jayanthan, Leonard Da Silva, Peter T. Simpson, Sunil R. Lakhani

**Affiliations:** ^1^ UQ Centre for Clinical Research, Faculty of Medicine The University of Queensland Brisbane Qld Australia; ^2^ Pathology Queensland The Royal Brisbane and Women’s Hospital Brisbane Qld Australia

**Keywords:** breast cancer, clonal progression, comparative genomic hybridization, E‐cadherin, metastasis, recurrence

## Abstract

Breast cancer metastasis to the stomach is rare; invasive lobular carcinoma has a predilection to spread to the gastrointestinal system and is morphologically similar to primary diffuse gastric carcinoma. This case highlights heterogeneous metastatic progression and that documentation of heterogeneity is important for informing future treatment strategies and prognostication.

## Graphical abstract

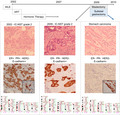

## 1 INTRODUCTION

The overall survival rate of patients with breast cancer has improved considerably over recent years. For patients whose tumor has metastasized, however, the outcome remains dismal with 5‐year survival rates of ~27%.^1^ A key area of breast cancer research is therefore trying to gain a clearer understanding of metastatic progression. Recent large‐scale molecular profiling initiatives (driven by consortia such as the International Cancer Genome Consortium [ICGC], The Cancer Genome Atlas [TCGA], and Molecular Taxonomy of Breast Cancer International Consortium [METABRIC]) to catalogue somatic alterations and gene expression profiles epitomize the extensive diversity within breast cancer.^2^, ^3^, ^4^ As powerful as these approaches are, the analyses underestimate the extent of intratumor heterogeneity within an individual, which has been elegantly illustrated in studies performing multiregion genome sequencing of matched primary tumor and metastatic deposits from the same patient.^5^, ^6^, ^7^, ^8^, ^9^, ^10^, ^11^ This demonstrates how dynamic clonal evolution may be within a tumor and that the ‘lethal’ clone may exist within a small and specific region of the primary tumor. It is impractical to consider multiple sampling of the primary tumor for sequencing applications in routine clinical practice, yet there is scope to appreciate and document intratumoral heterogeneity within a primary tumor^6^ to help develop our understanding of clonal evolution and metastatic progression.

To illustrate this, we present a case of invasive carcinoma of no special type (IC‐NST) from a patient who developed a local recurrence and widespread metastatic disease of the gastrointestinal tract with a range of histological patterns and immunophenotypes. Different tumor components were studied using comparative genomic hybridization (CGH) to detect DNA copy number changes in an attempt to understand clonal relationships as well as to highlight the challenges that this can create for the pathologists and oncologists in their routine practice.

## 2 CLINICAL HISTORY

A 52‐year‐old female patient underwent a wide local excision of a 13‐mm left breast lesion in 2002. A histological grade 2 invasive carcinoma of no special type (IC‐NST) with clear margins was diagnosed. The tumor was positive for estrogen (ER) and progesterone (PgR) receptors and negative for HER2. Adjuvant therapy consisted of radiotherapy (61.1 Gy in 30 fractions over 6 weeks) followed by Tamoxifen, which was changed to Arimidex due to endometrial thickening. Hormonal therapy ceased in 2007. In 2009, a 6 mm recurrence of histological grade 2 IC‐NST was detected at the site of the 2002 surgery; mastectomy and axillary clearance were carried out and followed by four cycles of Docetaxel/Cyclophosphamide. In the same year, a biopsy of the stomach identified a malignancy and a subsequent subtotal gastrectomy was performed revealing infiltrating adenocarcinoma of the distal stomach resection and omentum but not of the large bowel or regional lymph nodes. The diffuse growth pattern of the carcinoma observed on surgical resection slides indicated a poorly cohesive gastric carcinoma or a metastasis arising from an undiagnosed primary invasive lobular breast cancer. This diagnosis was followed by chemotherapy, which consisted of three cycles of Doxorubicin and Cyclophosphamide. The patient died in 2010.

### 2.1 Pathology review

The original classification and description of tumor morphology was reassessed and confirmed for all of the specimens. An associated intermediate‐grade DCIS component was also noted in the 2002 primary tumor. In the 2009 local recurrence, an IC‐NST with an associated component of intermediate‐grade and high‐grade DCIS was confirmed. The tumor in the stomach and omentum from 2009 showed a diffuse carcinoma infiltrating the whole thickness of the gastric wall as sheets of single cells and single files of cells, indicative of a metastatic lobular carcinoma of the breast or a poorly cohesive gastric carcinoma.

### 2.2 Immunohistochemical analysis

**FIGURE 1:**
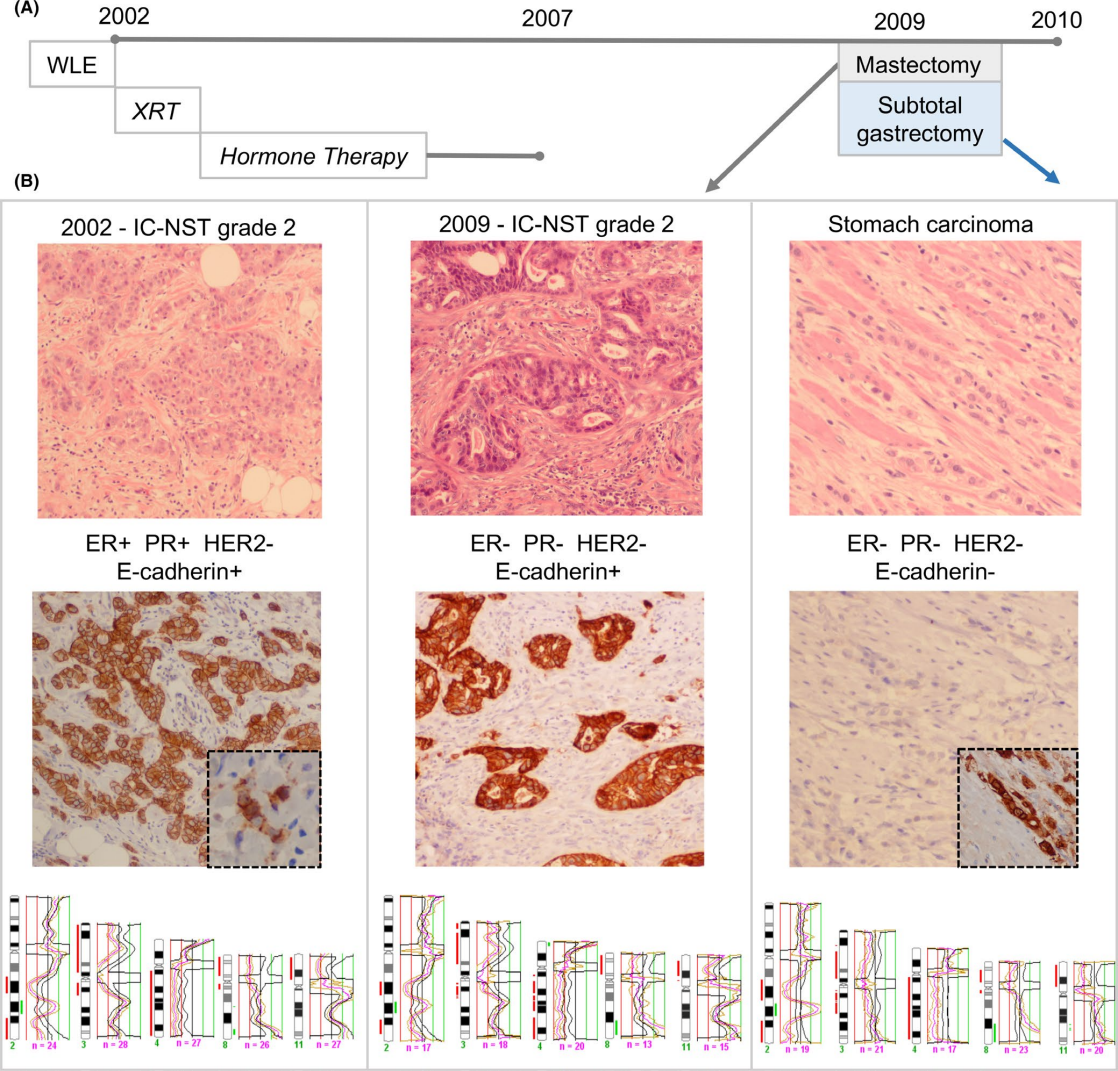
Overview of clinical, morphological, and molecular features. A, Timeline: the patient was initially diagnosed with breast cancer in 2002 following wide local excision (WLE) and received radiotherapy (XRT) and hormonal‐based therapy until 2007. In 2009, she underwent mastectomy for a recurrence and a subtotal gastrectomy for adenocarcinoma in the stomach. B, Morphological review of surgical resection slides revealed a grade 2 invasive carcinoma NST (10× magnification) in 2002 and in 2009 (10× magnification) and an infiltrative adenocarcinoma in the stomach (20× magnification) and omentum (not shown). The ER, PgR, and HER2 immunophenotype of each tumor is shown, together with E‐cadherin positive staining of the 2002 primary tumor (10× magnification) with a small population of single cells or single files of cells with aberrant staining (inset, 20× magnification). E‐cadherin staining in the 2009 breast tumor was normal (10× magnification), yet was completely absent in the stomach/omentum tumor (20× magnification). Strong aberrant cytoplasmic p‐120 catenin staining is also shown (inset, 20× magnification), reflecting altered cadherin‐catenin complex function. Comparative genomic hybridization shows the three tumors were clonally related with the same pattern of copy number alterations. Chromosome profile for chromosome 2, 3, 4, 8, and 11 is shown

Immunohistochemistry data are summarised in Table 1 and Figure 1 (please refer to Table A1 in Appendix 1 (Materials and Methods) for staining conditions and scoring). In brief, the 2002 tumor and DCIS were ER (3+ in 100% of cells) and PgR (3+ in <5% of cells) positive and negative for HER2, whereas the 2009 local recurrence and the stomach/omentum carcinoma were triple negative (ie negative for ER, PgR, and HER2). The 2009 primary breast tumor recurrence was positive for basal markers EGFR (1+, 40% of cells) and CK5/6 (2+, 10% of cells), whereas both the 2002 breast tumor and the stomach/omentum tumors were negative. Immunohistochemical staining of the E‐cadherin adhesion complex (including β‐catenin and p‐120 catenin) was performed as loss of E‐cadherin and β‐catenin, and aberrant staining of p‐120 catenin are hallmarks of an invasive lobular phenotype. The 2002 and 2009 breast tumors exhibited normal distribution of E‐cadherin, β‐catenin, and p‐120‐catenin, whereas the stomach/omentum lesions were negative for E‐cadherin (Figure 1) and β‐catenin, and stained aberrantly for p‐120‐catenin. Interestingly, we observed a small cluster of cells in the 2002 tumor that appeared to grow in a single‐cell file and exhibit aberrant E‐cadherin staining (Figure 1B, inset). The mesenchymal marker vimentin and the transcription factor Snail were analyzed to determine whether E‐cadherin downregulation could be related to acquisition of a (partial) epithelial to mesenchymal phenotype. Both markers were negative in the neoplastic epithelium of all tumors analyzed. Staining for CK7 and CK20 was performed to further discriminate the origin of the stomach lesion. The neoplastic cells were CK7 positive (3+ in 80% of tumor cells) and CK20 negative, indicating that the tumor is likely to be a metastasis and not a primary gastric carcinoma.^12^, ^13^

**Table 1:** Immunohistochemistry and comparative genomic hybridization data

	Lesion (year)					
	IC‐NST (2002)	Intermediate‐grade DCIS (2009)	High‐grade DCIS (2009)	IC‐NST (2009)	Stomach (2009)	Omentum (2009)
ER	3 + 100%	0	0	0	0	0
PR	3 + 5%	0	0	0	0	0
HER2	0	0	0	0	0	ND
EGFR	0	0	0	1 + 40%	0	0
CK5/6	0	0	0	2 + 10%	0	0
CK14	0	0	0	0	0	0
CK7	ND	ND	ND	ND	3 + 80%	ND
CK20	ND	ND	ND	ND	0	ND
E‐cadherin	+/Ab	+	+	+	0	Ab
β‐catenin	+	+	+	+	0	Ab
p‐120‐catenin	+	+	+	+	Ab	Ab
Vimentin	0	0	0	0	0	ND
Snail	0	0	0	0	0	ND
Shared CNA	2q−/+/−, 3p−, 3q−, 4q−, 8p−, 8q+, 11p−, 20q+					ND
Unique CNA	*5p+*	*13q+*	5p−	*5p+*	*12p+*	ND
	14q−	21q+	*17q+*	*12p+*	14q+	
	*17q+*			*13q+*		
				*17q+*		

Note

Highlighted in italic are CNA shared between some, but not all, of the lesions.

Abbreviations: −, loss; +, gain; +, positive; 0, negative; Ab, aberrant; CNA, copy number alterations; DCIS, ductal carcinoma in situ; IC‐NST, invasive carcinoma of no special type; ND, no data.

### 2.3 Comparative genomic hybridization (CGH)

Comparative genomic hybridization was employed (see Appendix 1 for methodology) to identify copy number alterations and define the clonal relationships between the lesions. The lesions were small or diffusely infiltrated stroma and so were laser‐capture microdissected prior to analysis. The five different components harbored the same pattern of gross DNA copy number alterations (Table 1, Figure 1). Of note was a striking alteration on chromosome 2q, showing a deletion/amplification/deletion pattern in each lesion analyzed. The following alterations were also identified in all lesions: loss on 3p, 3q, and 11p, and gain on 8q and, 11p, 20q; and all invasive tumors and the high‐grade DCIS also had loss on 4 and 8p.

## 3 DISCUSSION

This patient developed IC‐NST in 2002 and was diagnosed with a recurrence at the same site 7 years later. In that year, she was diagnosed with malignancy in the gastrointestinal tract, for which the differential diagnosis was primary diffuse gastric cancer (now classified as poorly cohesive gastric carcinoma) or metastatic lobular breast cancer. Cancer genomics can clarify differential diagnoses such as this by defining relationships between the lesions in question. Ideally, high‐resolution genomics technologies would be applied; however, in this case, low‐resolution chromosomal‐based CGH analysis was employed due to the small and intricate nature of the lesions being examined, and the necessity for laser‐capture microdissection and DNA amplification. Nevertheless, this molecular analysis was sufficient to clearly indicate that all lesions examined were clonally related with a close degree of overlap in copy number aberrations. Thus, the 2009 breast lesion, encompassing DCIS and IC‐NST, appeared to be a recurrence of the original breast tumor, and the stomach tumor is a metastasis derived from one of the two previously diagnosed breast tumors, notwithstanding the clonal expansion of the phenotypical divergence to an invasive lobular‐like pattern of growth.

The current case illustrates the concept of intratumoral heterogeneity and clonal evolution during progression, where a switch in immunophenotype was observed. The primary breast tumors were both classified as grade 2 IC‐NST, and the stomach/omentum metastatic deposits were characteristically of invasive lobular carcinoma type. We hypothesize that a small population (subclone) of ‘lobular‐like’ neoplastic cells within the primary tumor that went undetected (perhaps represented by the small cluster of E‐cadherin aberrant cells we identified in the 2002 tumor sample), disseminated, and seeded the metastases. The evolution of this infiltrative lobular growth pattern coincided with dysfunction of E‐cadherin‐catenin cell adhesion complex, which is observed by complete absence of the E‐cadherin, β‐catenin, and cytoplasmic p‐120‐catenin in the metastasis. We have previously shown that a lobular‐like phenotype can arise via a ductal‐like pathway of tumorigenesis, as exemplified by the mixed ductal‐lobular histological subtype,^14^ and metastasis from such mixed tumors can be of ductal and/or lobular type.^15^ The mechanism of E‐cadherin downregulation in this context is yet to be elucidated, however, the change in phenotype is unlikely to be driven by an epithelial to mesenchymal transition as the mesenchymal marker vimentin and the transcription factor Snail were both negative in each tumor, and evidence from our previous work suggests that EMT does not play a role in the downregulation of E‐cadherin in ILC.^16^

The immunophenotype of breast cancer is crucial for the selection of targeted therapy. The expression of ER and PgR is dynamically controlled and so are also prone to being downregulated during progression.^17^, ^18^ The patient in this report had an ER/PgR‐positive primary tumor and hence received hormonal‐based therapy for 5 years post diagnosis. Two years following the end of treatment, she was diagnosed with the second primary tumor and metastatic disease, both of which were ER/PgR negative. Discordance in hormone receptor status between primary and metastatic disease has been reported to occur in 18% of cases for ER, and 42% for PgR,^18^ and downregulation tends to occur in a nonrandom manner, particularly in breast metastases to the lung, liver, and bone.^17^ It is unusual for a primary lobular carcinoma to be ER negative, and it is tempting to speculate that in this scenario, the immunophenotypic drift observed was driven by selection against long term endocrine‐based treatment. In order to confirm these ideas, high‐resolution multiregion exome or whole‐genome sequencing should be performed, and evolutionary genetics algorithms (eg PyClone) would need to be applied; a proposal precluded by the limiting archival material available.

The gastrointestinal tract is not a common metastatic site for breast cancer (the incidence is reported to range from 0.3% to 6%^19^, ^20^, ^21^ but is a preferential metastatic haven for lobular carcinoma compared to other types,^15^, ^22^, ^23^, ^24^, ^25^, ^26^ with 64%‐75% of primary breast tumors in reported series being of the lobular morphology.^21^, ^27^, ^28^, ^29^ Metastatic disease arising in patients with primary mixed ductal‐lobular carcinomas can either be ductal or of a diffuse lobular type in these specific targeted organs,^15^ supporting our hypothesis that a minor lobular‐like subclone in the first primary tumor may be responsible for the disease progression. The differential patterns of metastatic spread between IC‐NST and ILC are presumably related to the tissue architecture and microenvironment of the target organs and the characteristic diffuse growth pattern of ILC. It has also been reported that patients diagnosed with ILC who develop metastatic disease are also more likely to have widespread disseminated disease than patients with IC‐NST.^30^, ^31^ Again, this is presumably related to the indolent nature of ILC and its diffuse growth pattern, in which disseminated tumor cells elicit little damage to host tissues and hence can go undetected for many years.

In summary, intratumor heterogeneity is a critical aspect of cancer biology, encompassing morphological, immunophenotypic, and genomic evolution as tumor cell clones adapt to local microenvironmental pressures, genomic instability, and treatment. This unique case illustrates the potential for tumors to undergo morphological evolution and phenotypic drift over time and highlights the challenges that this can create for the pathologists and oncologists in their routine practice. Stomach metastases are rare, and to our knowledge, this is the first case report describing a lobular‐like metastasis from a primary IC‐NST.

## CONFLICT OF INTEREST

The authors declare no conflict of interest.

## AUTHOR CONTRIBUTIONS

JRK: performed immunohistochemistry and data analysis, and contributed to writing the manuscript; AEMR: performed CGH and data analysis; AS: performed pathology review and immunohistochemistry analysis; JJ: performed immunohistochemistry; LDS: conceived the study and its design, carried out the pathology review and analysis of immunohistochemistry, and wrote the manuscript; PTS: conceived of the study and its design, carried out analysis, and wrote the manuscript; SRL: conceived the study and its design, provided clinical interpretation, and helped draft the manuscript; all authors contributed to study design, experimental work, and/or analysis and approved the final manuscript.

## ETHICS APPROVALS AND CONSENT

Human research ethics committees of The University of Queensland (ref. 2005000785) and The Royal Brisbane and Women's Hospital (2005/022) approved the study. The patient gave written consent for this work to be done.

## Data Availability

The datasets used and/or analyzed during the current study are available from the corresponding author upon reasonable request.

## Appendix 1

### MATERIALS AND METHODS

#### Pathology review and immunohistochemistry

Diagnostic slides of three surgical specimens (2002 breast; 2009 breast; 2009 stomach) were retrieved and reviewed using the current criteria defined by the World Health Organization classification for breast and gastric cancers by LDS and SRL{Board, 2019 #58;Lakhani, 2012 #26}.

Immunohistochemistry for various markers (Table A1) was performed using the MACH1 Universal HRP‐Polymer Dection kit (Biocare Medical, LLC) or the Ventana Optiview detection kit (Ventana Medical Systems).

**Table A1:** Antibody details and method of scoring

Marker	Clone ID, antibody dilution, supplier	Scoring method
ER	1D5, 1:40 dilution, Dako	>1%; percentage and intensity recorded
PgR	1A6, 1:100 dilution, Dako	>1%; percentage and intensity recorded
HER2	Herceptest, Dako	Herceptest
EGFR	31G7, 1:100 dilution, Invitrogen	Any positivity (membrane/cytoplasmic); percentage and intensity
CK5/6	D5/16B4, 1:300 dilution, Millipore	Any positivity; percentage and intensity recorded
CK14	LL002, 1:40 dilution, Novocastra	Any positivity; percentage and intensity recorded
CK7	OV‐TL 12/30,1:100 dilution Dako*	Any positivity; percentage and intensity recorded
CK20	Clone ks20.8, Leica	Any positivity; percentage and intensity recorded
P63	4A4, 1:25 dilution, Dako	Any positivity; percentage and intensity recorded
E‐cadherin	HECD1, 1:50 dilution, Zymed	Positive: intact membrane staining, Aberrant: fragmented membrane and/or cytoplasmic staining, Negative: no staining. As above
β‐catenin	17C2, dilution 1:100, Novocastra	
p‐120‐catenin	98/pp120, 1:200 dilution, BD Transduction Labs	
Vimentin	V9, 1:400 dilution, Dako	Any positivity; percentage and intensity recorded
Snail	SN9H2, 1:100 dilution, Cell Signaling Technology	Any positivity; percentage and intensity recorded

Note

Antigen retrieval—all citrate buffer (pH 6.0), 3 min pressure cooker at 105°C, except for E‐cadherin (EDTA buffer [pH 8.0] for 2 min in a pressure cooker at 105°C), CK7 (citrate 95°C for 30 mins), CK20 (Ventana CC1 20 mins), and EGFR (Chymotrypsin [pH 7.4] enzymatic antigen retrieval at 37°C for 10 mins).

#### Comparative genomic hybridization (CGH)

Five components (IC‐NST from 2002; intermediate‐grade DCIS, high‐grade DCIS and IC‐NST from 2009; stomach adenocarcinoma from 2009) from the case were laser‐capture microdissected prior to DNA extraction and analyzed by CGH as previously described{Simpson, 2005 #17}.

## References

[1] Sundquist M , Brudin L , Tejler G . Improved survival in metastatic breast cancer 1985–2016. Breast. 2017;31:46‐50. 27810699 10.1016/j.breast.2016.10.005

[2] Stephens PJ , Tarpey PS , Davies H , et al. The landscape of cancer genes and mutational processes in breast cancer. Nature. 2012;486(7403):400‐404. 22722201 10.1038/nature11017 PMC3428862

[3] Network, T.C.G.A. . Comprehensive molecular portraits of human breast tumours. Nature. 2012;490(7418):61‐70. 23000897 10.1038/nature11412 PMC3465532

[4] Curtis C , Shah SP , Chin SF , et al. The genomic and transcriptomic architecture of 2,000 breast tumours reveals novel subgroups. Nature. 2012;486(7403):346‐352. 22522925 10.1038/nature10983 PMC3440846

[5] Casasent AK , Schalck A , Gao R , et al. Multiclonal invasion in breast tumors identified by topographic single cell sequencing. Cell. 2018;172(1‐2):205‐217 e12. 29307488 10.1016/j.cell.2017.12.007 PMC5766405

[6] Caswell‐Jin JL , McNamara K , Reiter JG , et al. Clonal replacement and heterogeneity in breast tumors treated with neoadjuvant HER2‐targeted therapy. Nat Commun. 2019;10(1):657. 30737380 10.1038/s41467-019-08593-4 PMC6368565

[7] Gerlinger M , Rowan AJ , Horswell S , et al. Intratumor heterogeneity and branched evolution revealed by multiregion sequencing. N Engl J Med. 2012;366(10):883‐892. 22397650 10.1056/NEJMoa1113205 PMC4878653

[8] Navin N , Kendall J , Troge J , et al. Tumour evolution inferred by single‐cell sequencing. Nature. 2011;472(7341):90‐94. 21399628 10.1038/nature09807 PMC4504184

[9] Savas P , Teo ZL , Lefevre C , et al. The subclonal architecture of metastatic breast cancer: results from a prospective community‐based rapid autopsy program "CASCADE". PLoS Med. 2016;13(12):e1002204. 28027312 10.1371/journal.pmed.1002204 PMC5189956

[10] Yachida S , Jones S , Bozic I , et al. Distant metastasis occurs late during the genetic evolution of pancreatic cancer. Nature. 2010;467(7319):1114‐1117. 20981102 10.1038/nature09515 PMC3148940

[11] Yates LR , Gerstung M , Knappskog S , et al. Subclonal diversification of primary breast cancer revealed by multiregion sequencing. Nat Med. 2015;21(7):751‐759. 26099045 10.1038/nm.3886 PMC4500826

[12] Chu P , Wu E , Weiss LM . Cytokeratin 7 and cytokeratin 20 expression in epithelial neoplasms: a survey of 435 cases. Mod Pathol. 2000;13(9):962‐972. 11007036 10.1038/modpathol.3880175

[13] Tot T . Patterns of distribution of cytokeratins 20 and 7 in special types of invasive breast carcinoma: a study of 123 cases. Ann Diagn Pathol. 1999;3(6):350‐356. 10594286 10.1016/s1092-9134(99)80013-6

[14] McCart Reed AE , Kutasovic JR , Nones K , et al. Mixed ductal‐lobular carcinomas: evidence for progression from ductal to lobular morphology. J Pathol. 2018;244(4):460‐468. 29344954 10.1002/path.5040 PMC5873281

[15] Lamovec J , Bracko M . Metastatic pattern of infiltrating lobular carcinoma of the breast: an autopsy study. J Surg Oncol. 1991;48(1):28‐33. 1653879 10.1002/jso.2930480106

[16] McCart Reed AE , Kutasovic JR , Vargas AC , et al. An epithelial to mesenchymal transition programme does not usually drive the phenotype of invasive lobular carcinomas. J Pathol. 2016;238(4):489‐494. 26510554 10.1002/path.4668

[17] Cummings MC , Simpson PT , Reid LE , et al. Metastatic progression of breast cancer: insights from 50 years of autopsies. J Pathol. 2014;232(1):23‐31. 24122263 10.1002/path.4288 PMC4288974

[18] Idirisinghe PK , Thike AA , Cheok PY , et al. Hormone receptor and c‐ERBB2 status in distant metastatic and locally recurrent breast cancer. Pathologic correlations and clinical significance. Am J Clin Pathol. 2010;133(3):416‐429. 20154280 10.1309/AJCPJ57FLLJRXKPV

[19] Menuck LS , Amberg JR . Metastatic disease involving the stomach. Am J Dig Dis. 1975;20(10):903‐913. 1190198 10.1007/BF01070875

[20] Oda I , Kondo H , Yamao T , et al. Metastatic tumors to the stomach: analysis of 54 patients diagnosed at endoscopy and 347 autopsy cases. Endoscopy. 2001;33(6):507‐510. 11437044 10.1055/s-2001-14960

[21] Taal BG , Peterse H , Boot H . Clinical presentation, endoscopic features, and treatment of gastric metastases from breast carcinoma. Cancer. 2000;89(11):2214‐2221. 11147591

[22] Arpino G , Bardou VJ , Clark GM , Elledge RM Infiltrating lobular carcinoma of the breast: tumor characteristics and clinical outcome. Breast Cancer Res. 2004;6(3):R149‐R156. 15084238 10.1186/bcr767 PMC400666

[23] Borst MJ , Ingold JA . Metastatic patterns of invasive lobular versus invasive ductal carcinoma of the breast. Surgery. 1993;114(4):637‐641; discussion 641–2. 8211676

[24] Harris M , Howell A , Chrissohou M , Swindell RI , Hudson M , Sellwood RA . A comparison of the metastatic pattern of infiltrating lobular carcinoma and infiltrating duct carcinoma of the breast. Br J Cancer. 1984;50(1):23‐30. 6331484 10.1038/bjc.1984.135 PMC1976917

[25] Jain S , Fisher C , Smith P , Millis RR , Rubens RD . Patterns of metastatic breast cancer in relation to histological type. Eur J Cancer. 1993;29A(15):2155‐2157. 8297656 10.1016/0959-8049(93)90053-i

[26] Sastre‐Garau X , Jouve M , Asselain B , et al. Infiltrating lobular carcinoma of the breast. Clinicopathologic analysis of 975 cases with reference to data on conservative therapy and metastatic patterns. Cancer. 1996;77(1):113‐120. 8630916 10.1002/(SICI)1097-0142(19960101)77:1<113::AID-CNCR19>3.0.CO;2-8

[27] Ayantunde AA , Agrawal A , Parsons SL , Welch NT . Esophagogastric cancers secondary to a breast primary tumor do not require resection. World J Surg. 2007;31(8):1597‐1601. 17578645 10.1007/s00268-007-9099-y

[28] McLemore EC , Pockaj BA , Reynolds C , et al. Breast cancer: presentation and intervention in women with gastrointestinal metastasis and carcinomatosis. Ann Surg Oncol. 2005;12(11):886‐894. 16177864 10.1245/ASO.2005.03.030

[29] Pectasides D , Psyrri A , Pliarchopoulou K , et al. Gastric metastases originating from breast cancer: report of 8 cases and review of the literature. Anticancer Res. 2009;29(11):4759‐4763. 20032432

[30] Ferlicot S , Vincent‐Salomon A , Médioni J , et al. Wide metastatic spreading in infiltrating lobular carcinoma of the breast. Eur J Cancer. 2004;40(3):336‐341. 14746850 10.1016/j.ejca.2003.08.007

[31] Rakha EA , El‐Sayed ME , Powe DG , et al. Invasive lobular carcinoma of the breast: Response to hormonal therapy and outcomes. Eur J Cancer. 2008;44(1):73‐83. 18035533 10.1016/j.ejca.2007.10.009

